# Rheology of paste-like food inks for 3D printing: Effects of nutrient and water content

**DOI:** 10.1016/j.crfs.2024.100847

**Published:** 2024-09-27

**Authors:** Z.Y. Bugday, A. Venkatachalam, P.D. Anderson, R.G.M. van der Sman

**Affiliations:** a Processing and Performance, Eindhoven University of Technology, Eindhoven, Netherlands; b Food Process Engineering, Wageningen University, Wageningen, Netherlands; c Wageningen Food and Biobased Research Center, Wageningen, Netherlands

**Keywords:** 3D food printing, Personalized nutrition, Rheology, Pea-based

## Abstract

This research delves into understanding the effects of composition on the rheological response of multi-component food inks for 3D food printing. Accordingly, the motivation is to decouple the nutrient and water content effects on the rheology. We formulated inks by combining pea fractions with water and employing a water-holding-capacity based hydration method. Rheology is characterized by steady shear rate and oscillatory strain amplitude sweeps. Strain sweep curves infer that the deformation response of all inks follows a similar trend, and samples sharing the same macronutrient formulation are mapped to a master curve after scaling with the elastic plateau modulus. Samples sharing the same macronutrient formulation mapped to a master curve after scaling with the elastic modulus. Shear rate testing showed that the inks were shear thinning yield stress materials. Shear rate sweeps also collapsed on a master curve scaled by the yield stress and critical shear rate on the y and x axes. The yield stress and the plateau modulus appeared to be controlled by the water content, while the shear and strain thinning exponents were independent of the formulations, inferring that the rheology is scaled by the water content while preserving the shear thinning response. Observing the independence of the rheological properties from the nutrient composition and scalability of the rheology by the water content provided a step forward in developing formulations with various nutrient content at desired ow properties, which promises personalized nutrition. Furthermore, the study shows the applicability of various rheological techniques, which are expected to contribute to the literature on the rheology of granular pastes.

## Highlights

• Rheology is controlled by pregelatinized starch, independent of nutrient composition.
• Inks with similar rheology are achieved through hydration based on water holding capacity.
• Ink firmness can be adjusted without changing the strain or shear rate response.
• The yield stress is correlated with the linear elastic modulus.

## Graphical abstract

**Figure:**
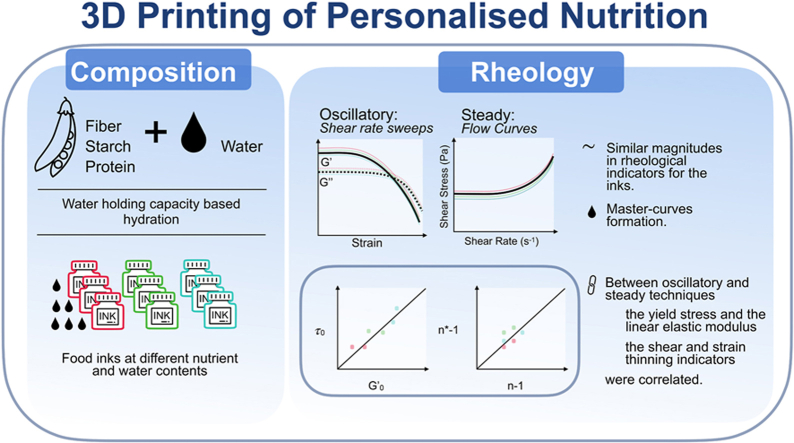

## 1 Introduction

A recent development in food processing is three-dimensional food printing (3DFP), which enables fully digitized food production while providing freedom in terms of shape, texture, and nutritional design (Liu et al., 2017; Jiang et al., 2021). This promises a combination of various ingredients to produce appealing products for different needs and purposes. For instance, 3DFP can produce personalized nutrition for the diverse requirements of specialized groups such as athletes, soldiers, dysphagia patients, and hospitalized people. Also, 3DFP offers a great solution to achieve food waste valorization by printing appealing shapes with the inks containing upcycled ingredients often rich in fiber (Pant et al., 2023; Uranga et al., 2023). Candy, pasta, and chocolate are typical, successful examples of printed foods (Yang et al., 2017; Lipton et al., 2015).

Extrusion-based 3DFP is an application of the Direct-Ink-Writing (DIW) technologies with customized food inks (Wei et al., 2023). It utilizes a simple deposition process where the food ink is pushed by a motor-driven force at a desired speed from a cartridge, extruded from a nozzle, and deposited layer-by-layer on a substrate. The coordinated movement of the substrate and the nozzle allows for creating targeted designs (Yang et al., 2017). Recent extrusion-based 3DFP studies endeavor to successfully print protein and fiber-rich formulations (Xie et al., 2022; Liu et al., 2022; Chen et al., 2022; Johansson et al., 2022; Zhang et al., 2022), various paste and gum formulations (Oyinloye and Yoon, 2022; Yan et al., 2022; Wei et al., 2021), dough preparations (Matas et al., 2022) and meat analogs (Yang et al., 2022; Wang et al., 2022).

Successful prints with DIW require the food ink to possess specific rheological characteristics. Inks should display fluid-like properties during the extrusion stage. They need to flow smoothly at the desired speed within the motor capacity of the printer. In addition, inks must build solid-like characteristics after extrusion to allow self-supporting structure, avoiding buckling or sagging, and adopt shape fidelity by reaching the target design (Zhang et al., 2018; Wei et al., 2023). The solidification mechanisms currently employed in 3DFP are the phase transition and yield stress recovery. In phase transition, the sample goes from a liquid state during extrusion to a solid state after deposition utilizing gelation or crystallization (Maldonado-Rosas et al., 2022; Liu et al., 2020; Kumbár et al., 2021). In the yield stress recovery mechanism, inks have a transient structure that undergoes a recoverable rearrangement when disrupted by shear and extensional flow during extrusion (Venkatachalam et al., 2023; Johansson et al., 2022). We focus on the yield stress mechanism in the scope of this work.

The rheological description of the yield stress ink in DIW printing involves an elastic nature under no deformation and a shear-thinning character during extrusion, showing elastic, plastic, and viscous properties (Kamani et al., 2021; Wei et al., 2023). In recent years, numerous studies have utilized different rheological techniques and parameters to understand the printing behavior of food inks. Lille et al. (2018), found that for a multi-nutrient paste formulation, ease of extrusion is linked to the plateau modulus (*G*_0_) while printing shape stability is connected to the deviation stress from the linear region in an oscillatory stress sweep test (Lille et al., 2018). Liu et al. (2019) concluded that the crossover point of storage (*G*′) and loss moduli (*G*″) (oscillatory stress sweep) was crucial during extrusion, as well as the shear thinning index described by a power law exponent from the viscosity measurement. Unlike the qualitative evaluation of composition, rheology, and printability, Zhu et al. (2019) obtained a quantitative correlation between the stress at collapse during printing and the flow stress (defined as the crossover point of storage and loss moduli in stress sweep) for tomato-based pastes (Zhu et al., 2019). Maldonado-Rosas et al. (2022) built upon Zhu et al. (2019) work and established a correlation between crossover moduli from oscillation amplitude sweep and printing quality indicators (Maldonado-Rosas et al., 2022). Lastly, Gholamipour-Shirazi et al. (2019) connected the ratio of loss to storage modulus in the linear region and relaxation exponent with the self-supporting properties of food hydrocolloid pastes (Gholamipour-Shirazi et al., 2019). Further, they stated a correlation between the phase angle and the strain softening coefficient (obtained from the *G*′ in a strain amplitude sweep).

Previous literature research shows that there is a growing consensus on the relationship between rheology and printability, besides a comprehensive understanding of the interplay between composition and its effects on ink rheology (Wei et al., 2023). Effect of various nutrients on the rheology is essential while targeting customization of the ink formulation. Our perspective is that rheological properties can ideally be utilized to decouple the functionality of various macronutrients and water content on ink behavior. Accordingly, previous studies on the elaborate investigation of composition and rheological properties enlightened important findings. For instance, van der Sman et al. (2022b), studied the rheological behavior of aqueous concentrated maltodextrins and starch systems, and showed that the glass transition temperature (normalized by measurement temperature: T_g_/T) governs the zero shear viscosity and shear thinning properties. Further, they showed that this approach is used to modulate the plasticizer content to achieve a targeted structure with the desired sugar content (van der Sman et al., 2022a). Similarly, they studied plant protein dough rheology and discovered that while the T_g_/T again appears as a scaling factor for the linear viscoelastic (LVE) regime, the oscillatory and steady rheological indicators were correlated and can be translated to each other (van der Sman et al., 2023a). Furthermore, they took a broader point of view and investigated the universal rheological response of paste and purees, developing a constitutive model that can accurately predict the strain sweep response. In addition, Venkatachalam et al. (2023), studied the effects of macronutrient composition directly on the printability and building characteristics of the pea-based complex food inks (Venkatachalam et al., 2023). They adjusted the water content according to a ratio of the total water holding capacity (WHC) of dry ingredients. Hypothesizing that the printability can be expressed as a function of the WHC of the ingredients. Further, they showed extrusion force, and the buildability was controlled by the macronutrient composition. Similarly, Johansson et al. (2022) studied the rheological and printing properties of starch, protein, and fiber-rich food inks from faba beans. They showed that storage and loss moduli and printing pressure were dominated by the composition (Johansson et al., 2022).

Recent studies clarified that each macronutrient brings different functionalities into the system, which can affect the rheology, see van der Sman et al., (2024). Herein, we aim to uncover the effect of water content and nutrient composition on the ink rheology, considering the customization of the ink formulation for personalized nutrition. We formulated paste-like pea-based-inks of varying compositions and water content by combining different fractions (starch, protein, fiber) with water. A WHC-based approach is adopted for hydration to test whether the rheology is scaled as a function of the WHC of the system. Preliminary work showed that the materials mentioned above are poor network formers, hence the water can easily move within the ink, causing undesired phase separation. This is prevented by adding a hydrocolloid, namely, pregelatinized pea starch, in the spirit of the approach. Hence, the inks are pictured as dispersed fiber, protein, and raw starch particles in a transient matrix of gelatinized starch. We evaluated the properties of food inks in terms of their steady and oscillatory rheological attributes. The motivation is to first discover the general trends and scaling relations in rheology with changing formulation. Then to elucidate the relationship between oscillatory and steady rheological indicators for translation from one to another.

## 2 Materials and methods

### 2.1 Food ink preparation

#### 2.1.1 Materials

The food inks described in this study were prepared using pea fractions and water. Specifically, we used pea fractions containing pregelatinized (PG) starch (Nastar Precooked Pea Starch), raw starch (Nastar Native Pea Starch), native (partially denatured) protein (Pisane M9 Pea Protein), and insoluble fiber (Swelite C Pea Fiber), which were kindly provided by Cosucra Groupe Warcoing SA in Belgium. All the raw materials were in powder form.

#### 2.1.2 Raw material characterization

The materials were tested for moisture content (MC) and WHC. The measurement of the MC was based on a weighing method before and after complete drying. Furthermore, the WHC of pure ingredients was measured based on analyzing the residual water on the soaked samples. Following soaking, the sample was centrifuged and the residual moisture was analyzed by weighing before and after complete drying. For a full description of the methods, we refer to (Venkatachalam et al., 2023). The measured reference values for this study are provided in Table 1.

**Table 1:** Reference values for the moisture content and the water holding capacity of ingredients.

Substance	Moisture Content g water/g substance	Water Holding Capacity g water/g dry solids
PG Pea Starch	0.10	5.05
Raw Pea Starch	0.10	11.0
Pea Protein	0.04	1.05
Pea Fibre	0.10	12.1

#### 2.1.3 Formulation and ink preparation

Pea fractions were combined in various ratios on a dry basis to obtain printing inks, as detailed in Table 2. The “rich” inks were the main sample set used to validate the WHC hypothesis, test the experimental protocols and sample preparation technique. Following the “rich” groups, “mid” and “low” samples were contributed to study the effects of changing fiber, starch, and protein content.

**Table 2:** Weight ratio of the nutrients on a dry basis. All the samples contained PG starch at 1:10 ratio of PG starch:total dry mass.

Sample	Raw Starch	Fibre	Protein
Protein Rich^a^	1	1	3
Fibre Rich^a^	1	3	1
Starch Rich^a^	3	1	1
Protein Mid^b^	3	3	4
Fibre Mid^b^	3	4	3
Starch Mid^b^	4	3	3
Protein Low^b^	2	2	1
Fibre Low^b^	2	1	2
Starch Low^b^	1	2	2

The batch size was 100g for each sample preparation.

a Hydrated at 45, 50, 55% WHC.

b Hydrated at 50% WHC.

The dry fraction was hydrated based on a fraction of the WHC of the mixture, as calculated based on Equation (1).

(1) $$m_{\text{water}}=p\left(\overset{N}{\underset{n}{\sum }}M_{n}{WHC}_{n}-\overset{N}{\underset{n}{\sum }}M_{n}X_{n}\right)$$

*m*_water_ is the amount of added water (kg), *M*_*n*_ and *X*_*n*_ were the dry mass (kg dry basis) and water content (kg water/kg db) of an ingredient n. Note that dry matter contains a small amount of moisture absorbed from the surroundings. Finally, *N*=[fibre, protein, starch, PG starch] and *p* is the percentage of the WHC of dry fraction, respectively. For this study, we selected hydration percentages, *p*, of 45%, 50%, and 55% to hydrate the fiber, starch, and protein-rich compositions at 3 different water levels. The selected water levels were decided through preliminary printing experiments to achieve a flowing structure and self-supporting strength while avoiding visible phase separation. The square geometry mentioned in Venkatachalam et al. (2023) is printed to test the inks. We want to highlight that the interactions between the nutrients and the particle size of raw materials can affect the WHC of the systems (Lee et al., 2021). Accordingly, we expect PG starch to capture the free water and prevent any inconsistency in the ink properties that can arise from the mobility of free water.

The dry ingredients were weighed and mixed in a beaker until they formed a homogeneous mixture and then introduced into the water at room temperature. After stirring with a spoon for 30 s, total mixing was ensured by using a conventional mixer (Kenwood Kitchen Mixer, UK) with a flat mixing hook for 5 min. By mixing, all the materials were equally distributed, and the pregelatinized starch was prevented from forming big pieces of hydrogen-bonded clusters. Following preparation, the samples were deaerated using a centrifuge vacuum mixer (Thinky Vacuum Mixer ARV-310LED, USA) under 30 kPa and at 2000 rpm, at 25C for 2 min. No water loss of phase separation is observed during the vacuum degassing stage. Finally, samples were sealed to prevent evaporation and allowed to hydrate overnight at room temperature to reach equal moisture distribution over all ingredients. Samples were tested on the following day.

### 2.2 Rheological measurements

Strain amplitude and shear rate sweeps were utilized to measure the composition-dependent rheological properties of 3DP inks. Measurements were performed using an Anton Paar MCR 501 Rheometer (Anton Paar GmbH, Austria) at 25 °C. This rotational rheometer was used for strain amplitude or shear rate-controlled experiments, with measurements of shear stress, and storage (*G*′) and loss (*G*″) moduli. Both measurements were conducted using a 50 mm diameter stainless steel plate-plate measurement system with a crosshatched surface (PP50/P2). The prior aim of selecting a crosshatched plate was to prevent the wall slip by incorporating maximum roughness with the profiles on the surface of the plate. A sample of 5 g was placed on the bottom plate of the rheometer, and the gap height was adjusted to 1 mm for measurement. Rheological measurements of “rich” groups were replicated for two different batches of the same preparation. Measurements of the “mid” and “low” groups were replicated for the testing protocol by samples from the same batch. The repeatability was examined by the coefficient of variation (COV) (standard deviation normalized by the mean) by setting a criterion, where the COV is less than 15%. The standard deviation between replicates was presented in the error bars for the data.

During preliminary experiments, we found that the post-loading residual normal force affected the measurements. Following loading, we waited 3 min at measuring height, without any imposed shear or strain rate, for the normal force to reach an equilibrium. Initially, the equilibrium value was typically between 5 and 30 N, so full relaxation was not observed. Therefore, specifically for the shear rate sweeps, where the residual normal force was observed to have more impact, we increased the gap height by a distance of 0.03–0.4 mm, targeting an initial normal force between 0 and 3 N, which ensures full contact between material and plates, and equal contribution of residual normal force on the measurement. We have confirmed by preliminary testing that the measurements were not dependent on the gap height; hence, the slip effects were eliminated.

#### 2.2.1 Strain amplitude sweeps

Amplitude sweeps were conducted from a strain amplitude of 0.01–1000% (applied in the increasing direction) at 1 Hz, with 10 data points collected per decade. Each data point was given a fixed time of 5 s, which ensured the steadiness of the values by completing 5 cycles. *G*′ and *G*″, are used to analyze the strain amplitude dependent structural changes during the amplitude sweep test for the whole amplitude range. *G*′ and *G*″ are obtained from the standard output of the rheometer.

The strain amplitude dependency of the viscoelastic moduli is evaluated by fitting *G*′ and *G*″ to a descriptive model, given in Equations (2), (3)).

(2) $$\begin{array}{lll} \frac{G^{\prime }}{G_{0}}={\left(1+{\left(\frac{\gamma }{\gamma_{\text{cr}1}}\right)}^{a}\right)}^{\left(n_{1}-1\right)/a}, & & \end{array}$$

(3) $$\begin{array}{lll} \frac{G^{\prime \prime }}{G_{0}\text{tan}\left(\delta \right)}={\left(1+{\left(\frac{\gamma }{\gamma_{\text{cr}2}}\right)}^{a}\right)}^{\left(n_{2}-1\right)/a}. & & \end{array}$$

Where *γ*_cr1_ and *γ*_cr2_ indicate the critical strain amplitude defining the extent of the linear region. More specifically, the critical strain amplitudes indicate the cross-section strain of the two power-law models describing the linear and strain softening region as mentioned in Dinkgreve et al. (2016). *n*_1_ and *n*_2_ are the strain softening exponents, and *a* is a fitting constant. *G*_0_ is the plateau modulus and tan(*δ*) = *G*″/*G*_0_ in the linear region. By using the following model, strain amplitude at the decay from linearity for both *G*′ and *G*″ was quantified separately by *γ*_cr1_ and *γ*_cr2_. Similarly, the strain-amplitude-dependent decay was expressed by *n*_1_ and *n*_2_ in the larger strain amplitude regions.

*G*_0_ and tan(*δ*) are obtained by averaging the first 5 data of each set. The standard deviation from the average data set involved was reported as the error in *G*_0_ and tan(*δ*). The model parameters *γ*_cr1_, *γ*_cr2_, *n*_1_, and *n*_2_ were estimated by the least square fitting method for different values of *a*, from Equations (2), (3)), between 0.8 and 1.5. The quality of fitting is ensured by the residual sum of squares (*R*^2^) greater than 0.99 and the fitting parameters with the highest *R*^2^ were selected. The variance in the estimated parameters was reported as an error. It is obtained by the squared sum of the diagonal elements of the covariance matrix calculated by the “*curve fit*” function from the Python module Scipy (More et al., 1980).

Finally, the crossover strain (*γ*_x_) was calculated from the data to be the strain amplitude with a minimal difference between the *G*′ and *G*″. Error is reported as the standard deviation from the means of data sets involved in the calculation.

#### 2.2.2 Shear rate sweeps

Shear rate sweeps were performed by collecting 5 data points per decade, with shear rates ranging from 0.1 to 500 1/s. The time allotted for each data point was logarithmically decreased from 60 to 1 s. It is important to state that during the preliminary experiments, inks demonstrated a hysteresis between increasing and decreasing shear rate testing. Accordingly, we declare incremental shear rate testing results in this study.

Experimental data is analyzed by fitting to a Herschel-Bulkley model given in Equation (4), by the least squares fitting where, *τ*_0_ is the yield stress, *n* is the shear thinning exponent and the ${\dot{\gamma }}_{\text{cr}}$ is the critical shear rate, which provides dimensionless interpretation (Morrison, 2001). The Herschel-Bulkley model is selected since it is the simplest and the most widely used model to describe the flow curves with a plateau on the lower and a power law response on the higher shear rate regions (Wei et al., 2023). The variance in the fitted parameters was calculated by the squared sum of the diagonal elements of the covariance matrix calculated by the “*curve fit*” function from the Python module Scipy and reported as error bars:

(4) $$\frac{\tau }{\tau_{0}}=\left(1+{\left(\frac{\dot{\gamma }}{{\dot{\gamma }}_{\text{cr}}}\right)}^{n}\right).$$

### 2.3 Comparing the steady and oscillatory rheological indicators

The expression of the yield stress obtained by the steady shear and oscillatory shear rheology were compared to answer if two different techniques can measure comparable values of the yield stress for our materials. Considering that the flow curves were constructed in an ascending manner, we took the plateau value of the stress from the shear rate sweeps to be the yield stress. The plateau value is captured by the Herschel-Bulkley model parameter (*τ*_0_), described in detail in Section 2.2.2. Also, the strain sweeps were constructed from the low to high strain, similarly, to extract the yield stress from strain amplitude sweeps, we utilized the elastic description in small amplitude oscillatory flow:

(5) $$\tau =G\gamma_{el},$$

where *G* is the elastic modulus *γ*_*el*_ is the recoverable elastic strain amplitude satisfying the following relation: |*γ*_*el*_| < *γ*_*cr*_ (Doraiswamy et al., 1991). *γ*_*cr*_ is the critical strain amplitude indicating the end of the linear region and the yield stress from oscillatory experiments (*τ*_0,osc_) can be expressed as:

(6) $$\tau_{0,osc}=G_{0}\gamma_{cr}.$$

In the spirit of our approach, the curve shape characteristics were determined by descriptive model fitting (refer to equations (2), (3))); accordingly, we took *γ*_*cr*_ = *γ*_cr1_ and *G* = *G*_0_. It is important to clarify that equation (6) assumes that all the strain acquired until the yielding is elastic. However, recent evidence shows that this assumption might not be exact (Kamani et al., 2021). Since we are looking for a simple comparison of the yield stress values with this study, we hold the assumption.

For the large deformation region, the shear and strain thinning properties from the steady and oscillatory rheology are compared. From the steady rheology, the shear thinning exponent of the viscosity is evaluated. The shear thinning exponent (*n* − 1) from the Herschel-Bulkley fitting describes the shear thinning characteristics in steady shear according to equation (4), the viscosity expression of the Herschel-Bulkley model is provided in equation (7) for clarification.

(7) $$\eta \left(\dot{\gamma }\right)=\frac{\tau_{0}}{\dot{\gamma }}+\frac{\tau_{0}}{{\dot{\gamma }}_{\text{cr}}^{n}}\;{\dot{\gamma }}^{n-1}.$$

To investigate the strain thinning properties captured from the oscillatory rheology, the energy dissipation-dominated region above the crossover strain is evaluated via the loss modulus. Viscosity in that region is known to be expressed by:

(8) $$\eta_{\text{dis}}\left(\gamma \omega \right)=\frac{G''\left(\gamma \right)}{\omega }.$$

where *G*″ (*γω*) is the strain amplitude and frequency (*γ*, *ω*) dependent loss modulus, *ω* is the frequency and *η*_dis_(*γ*) is the energy dissipation region viscosity (Barnes, 2000). The change of *η*_dis_ with strain amplitude is evaluated by fitting the last 10 datapoints from the strain amplitude sweep (at strains being greater or equal to the crossover strain) to the shear thinning part of equation (7), as described in equation (9) (Doraiswamy et al., 1991),

(9) $$\eta_{\text{dis}}\left(\gamma \omega \right)=\frac{{G''}^{}\left(\gamma \right)}{\omega }∼\frac{\tau_{0}}{{\dot{\gamma }}_{\text{cr}}^{n}}{\left(\gamma \omega \right)}^{n-1}∼K^{*}{\left(\gamma \omega \right)}^{n^{*}-1}.$$

*n*∗ and *K*∗ are fitting constants, *n*∗ − 1 is compared with *n* − 1 extracted by the Herschel-Bulkley model fitting of the steady shear data.

## 3 Results and discussion

### 3.1 Strain amplitude sweeps

**Fig. 1:**
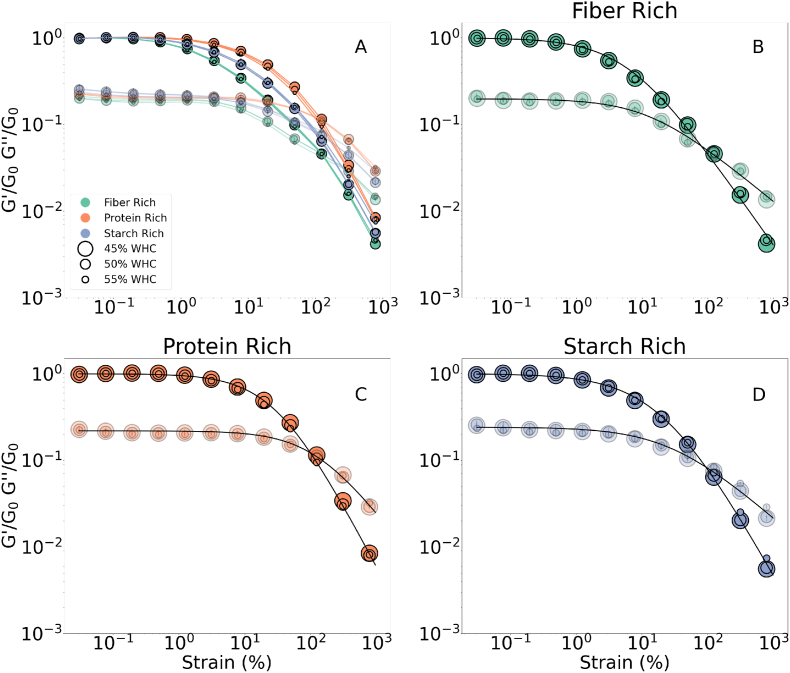
Strain amplitude sweeps for rich groups normalized over the *G*′ in linear viscoelastic region. Different formulations were indicated by colors and the hydration levels were indicated by the marker size. (For interpretation of the references to color in this figure legend, the reader is referred to the Web version of this article.)

**Fig. 2:**
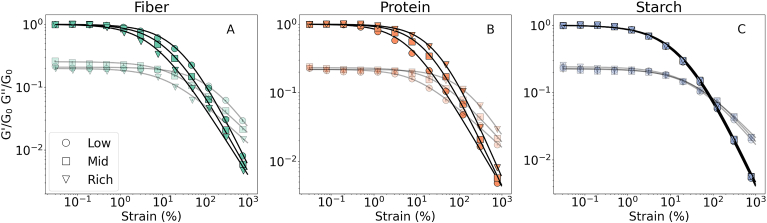
Strain amplitude sweeps for changing fiber, protein, and starch contents. All samples are hydrated at a level of 50% WHC.

Results of the strain amplitude sweep test were given in Fig. 1, Fig. 2 where the strain-amplitude-dependent changes were visualized by the *G*′ and *G*″ normalized over the plateau modulus, *G*_0_. Please note that once in every 4 datapoints is plotted in Fig. 1, Fig. 2 to keep a clear visualization of the data while conveying the critical messages. For all the samples, both *G*′ and *G*″ decreased with increasing strain amplitude and started to decay from different amplitudes. Sim et al. (2003) classifies this type of moduli (shown in Fig. 1, Fig. 2) behavior as the strain softening led by an increase in strain amplitude either causing structural rearrangement or loss of junctions within the transient network resulting in strain softening (Hyun et al., 2002). On the other hand, Kamani et al. (2021) utilizes a recoverable and unrecoverable dissipations approach to explain the curve shapes of storage and loss modulus, depending on how elastic and plastic strain is acquired. If the recoverable dissipation term is constant or decreases with increasing strain amplitude while the unrecoverable term is increasing, a longer plateau or an overshoot in loss moduli is observed compared to the storage modulus. Furthermore reaching higher in strain amplitude, a structural loss causes both moduli to decay. The latter approach better enlightens two distinct curve shapes from storage and loss modulus.

To investigate the effects of varying macronutrient composition and water content, tan(*δ*), crossover strain (*γ*_x_), and descriptive model parameters were evaluated (see eqns. (2), (3)) for the descriptive model details). The descriptive model data is pictured in Fig. 1 and all the estimated parameters are provided in Table 3.

**Table 3:** Curve shape indicators from the strain sweep curves.

Sample	tan(*δ*)∗ [-]	$\gamma_{\text{x}}^{+}$ [%]	$\gamma_{\text{cr}1}^{o}$ [%]	$\gamma_{\text{cr}2}^{o}$ [%]	$n_{1}^{o}$ [-]	$n_{2}^{o}$ [-]	*a* [-]
Fiber Low	0.21 ± 0.01	108 ± 12.4	29.1 ± 1.09	139 ± 9.46	−0.44 ± 0.02	−0.02 ± 0.03	0.9
Fiber Mid	0.25 ± 0.01	95.3 ± 0.02	13.6 ± 0.63	20.5 ± 1.37	−0.24 ± 0.02	0.33 ± 0.02	0.9
Fiber Rich	0.20 ± 0.01	134 ± 15.7	6.29 ± 0.25	13.4 ± 0.72	−0.09 ± 0.01	0.33 ± 0.02	0.9
Protein Low	0.21 ± 0.01	98.5 ± 0.00	6.27 ± 0.38	10.51 ± 0.62	−0.07 ± 0.02	0.43 ± 0.01	1.0
Protein Mid	0.23 ± 0.01	98.4 ± 0.01	17.2 ± 0.64	30.47 ± 2.22	−0.33 ± 0.02	0.29 ± 0.02	1.0
Protein Rich	0.22 ± 0.01	115 ± 11.9	31.7 ± 0.71	129 ± 7.38	−0.47 ± 0.01	−0.01 ± 0.03	1.0
Strach Low	0.23 ± 0.01	98.4 ± 0.01	19.4 ± 0.55	40.4 ± 1.84	−0.38 ± 0.01	0.22 ± 0.01	0.8
Strach Mid	0.22 ± 0.01	88.3 ± 10.1	20.5 ± 0.61	48.3 ± 2.74	−0.38 ± 0.01	0.21 ± 0.02	0.8
Starch Rich	0.24 ± 0.01	98.4 ± 0.06	21.9 ± 0.67	29.4 ± 2.29	−0.39 ± 0.01	0.33 ± 0.02	0.8

∗Error bars indicate the standard deviation between the first 5 data of the corresponding dataset.

+Error bars indicate the standard deviation between the different datasets.

oError bars indicate fitting errors.

For the rich groups, *G*_0_ normalization led to the formation of three typical master curves for each macronutrient combination, indicated by different colors in Fig. 1. Within each class, *G*_0_ appears to be water content dependent since different water contents of the same formulation collapse on a master curve. For the descriptive model fitting, each master curve is described by one set of model parameters, tan(*δ*) and *γ*_x_. For the mid and low groups, each sample was described by one set of parameters to quantify the difference between curve shapes.

We first focus on the macronutrient contributions to the master curves’ characteristics, and later discuss the effects of water content.

Starting from the lower strain amplitude region, firstly tan(*δ*) is evaluated. It appeared to be independent of the composition and all the samples showed an approximate value of 0.2–0.3, as given in Table 3. Materials with a tan(*δ*) greater than 1 are considered to be more viscous than elastic and vice versa (Steffe, 1996). Results revealed that the inks are more dominantly elastic than viscous within the limits of the LVE regime, independent of the formulation and the water content. Furthermore, they remain dominantly solid after their elastic critical strains (*γ*_cr1_). Having solid-dominated behavior for an extended strain amplitude region is considered an advantage for 3DFP inks since it helps to achieve accurate layer-by-layer building and shape fidelity. Furthermore, a strong structure after printing results in an easy hand-held material (Kim et al., 2017). We consider this behavior as a key property while achieving successful printable formulations.

Continuing along the strain amplitude range, we reach the critical strains: *γ*_cr1_ and *γ*_cr2_. Investigating the rich groups (Fig. 1), showed that the linear regime is prolonged in protein-rich inks followed by starch and fiber-rich. The same observation is also valid in the mid and low groups; the primary and secondary subfigures in Fig. 2 show that the extent of the LVE region increases with decreasing fiber content and decreases with the decreasing protein content, but appears independent of the starch content. This shows a clear difference between the deformation characteristics of the different macronutrient-dominated systems. We believe that the difference arises from the diverse roles of macronutrients in the microstructure which is discussed further in Section 3.4.

Further in the strain amplitude sweep plot, we reach the crossover point where the *G*′ and the *G*″ are equal to each other. Crossover strain, *γ*_x_ was distributed around 100% strain amplitude for all the samples (refer to Table 3). Crossover strain showed only a minor difference between different samples but neither with a pattern nor a change in the order of magnitude. Hence we conclude that it appeared to be independent of the macronutrient composition and the water content. The findings of (van der Sman et al., 2023a) support our observations, where the critical strain was found to be independent of the moisture content for the protein-rich formulations used in the meat analogs. Similarly, in a different study, they compared various strain amplitude sweep results of fruit/vegetable purees and found that the crossover point does not differ in various formulations (van der Sman et al., 2024). Furthermore, this phenomenon is also observed in aqueous Carbopol solutions generalized as concentrated microgels (Ketz and Graessley, 1988).

**Fig. 3:**
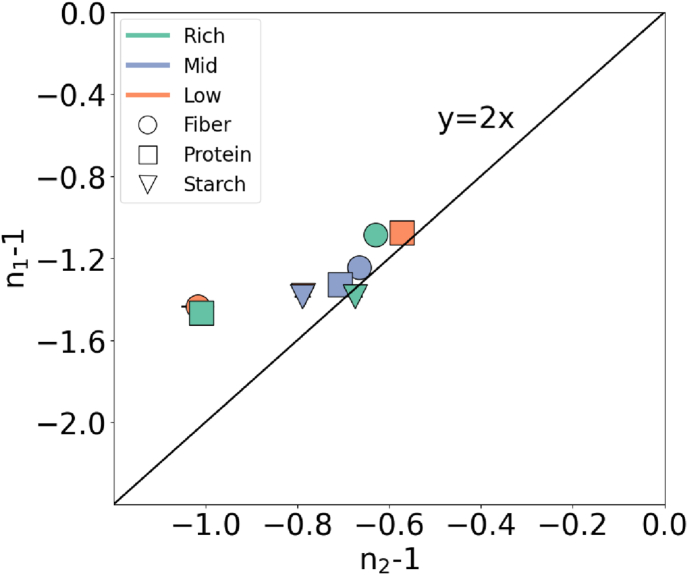
Relationship between strain softening indices of *G*′ and *G*″ namely *n*_1_ and *n*_2_.

The final common characteristic of all the strain amplitude sweep plots is a power law decaying region where the unrecoverable changes dominate and consequently, structural loss and strain softening are observed. The descriptive model provides the exponents to quantify the strain-softening effect (*n*_1_ and *n*_2_). The exponents appeared independent of the water content and the macronutrient ratio. Further, when plotted against each other, most of them appeared to be distributed around the *y* = 2*x* line, indicating a linear correlation by a factor of 2 (*n*_1_/*n*_2_ ∼ 2), as shown in Fig. 3. There were two eye-catching outliers, fibre-low and protein-rich inks. Both inks have a protein: fiber ratio ≥2 (refer to Table 2), possibly affecting the ratio of the recoverable and unrecoverable dissipation. The findings followed the patterns obtained for the aqueous protein dispersions for meat analogs and fibre suspensions. Previously it has been shown that the *G*′ ∼ *γ*^2(*n*−1)^ and *G*″ ∼ *γ*^(*n*−1)^, where n is their strain thinning exponent (van der Sman et al., 2023a, van der Sman et al., 2024).

An interesting observation in the macronutrient-driven curve shape characteristics was the response of systems with varying starch content. First of all, they were unresponsive to the changes in the starch concentration, as can be observed in Fig. 2(C) and Table 3. However, they showed different critical strains than the fiber and protein-rich samples while having a similar order of the crossover strain and a comparable softening exponent (refer to Table 3).

**Fig. 4:**
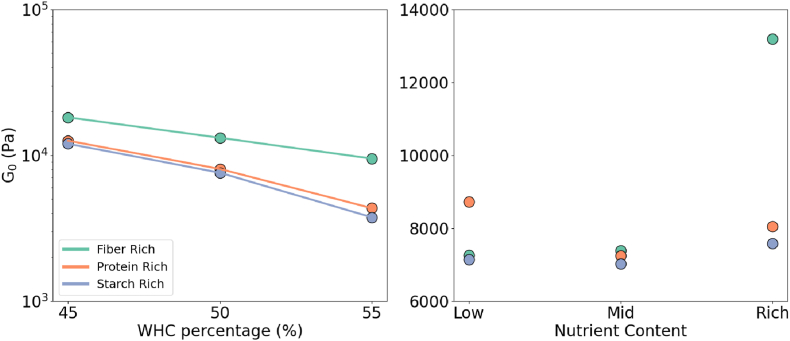
Change in the plateau modulus (*G*_0_) with respect to the water content and macronutrient content at 50 % WHC.

Focusing on the effects of changing water content and macronutrient ratio on the rheological properties, we see that they affect *G*_0_. Fig. 4 clearly displays that increasing water content results in a slight decrease in *G*_0_. Considering that the *G*_0_ holds as a vertical scaling factor for the strain amplitude sweep plots, it can be inferred that the water content appears as a scaling factor for the curves within the given composition range. Another important point is that *G*_0_ is stated to be a good indicator of the firmness (Lille et al., 2018) for the 3DFP inks; we see that it is possible to manipulate the firmness of the sample with water content while keeping the unique strain-softening properties introduced by the macro-nutrient unchanged. This further extends to the texture of the printed food material, where the textural modifications become apparent with the water content. The effects of nutrient content at a constant WHC ratio (50%) show that the fiber content has a strong effect on the *G*_0_, while starch content lightly controls and protein content does not seem to cause a trend.

### 3.2 Shear rate sweeps

**Fig. 5:**
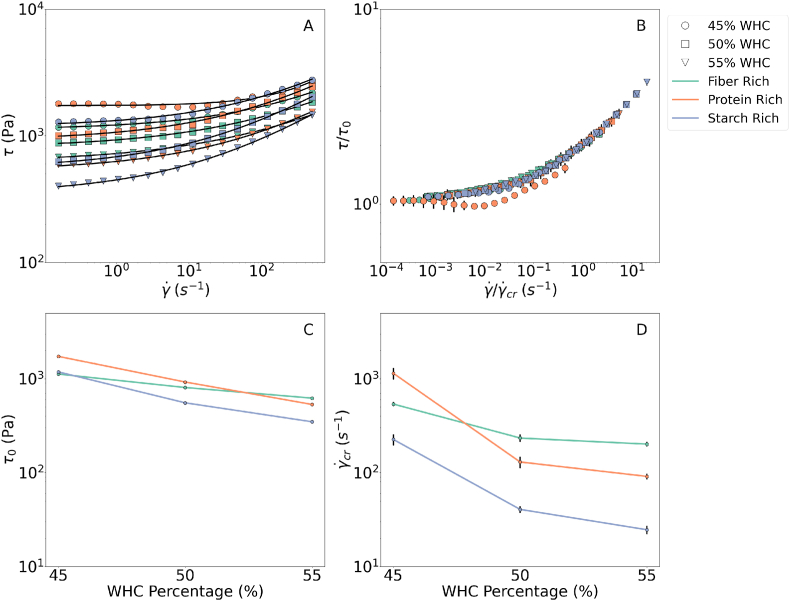
Results of the shear rate sweep test. Different colors indicate macronutrient formulations and markers indicate water contents. (1) shows the shear rate shear stress relationship with the fitted Herschel-Bulkley function with the black curves. (2) The shear-rate sweep data normalized over the $\dot{\gamma_{\text{cr}}}$ and *τ*_0_. (3) and (4) the *G*_0_ and the *τ*_0_ with changing water content. (For interpretation of the references to color in this figure legend, the reader is referred to the Web version of this article.)

Results of the shear rate sweeps were given in Fig. 5(A) for fiber, starch, and protein-rich inks, with the colored markers.

The shear rate-shear stress data is fitted to a Herschel-Bulkley model (Equation (4)). The model data is given on the same plot with the black line model parameters and the fitting parameters were provided in Table 4. Note that Fig. 5(B) is normalized over the fitting parameters. As a result, a master curve is obtained indicating that the shear thinning exponent *n* appears to be independent of the macronutrient composition, as can be seen in Table 4.

**Table 4:** Power law exponents from theoretical and experimental flow data.

Sample	Hydration	*τ*_0_	${\dot{\gamma }}_{\text{cr}}$	*n*
Fibre Rich	45	1119 ± 11.71	536.8 ± 26.37	0.391 ± 0.014
	50	805.1 ± 11.63	233.1 ± 20.30	0.347 ± 0.011
	55	620.5 ± 5.306	201.1 ± 10.76	0.339 ± 0.006
Protein Rich	45	1727 ± 24.64	1143 ± 162.9	0.714 ± 0.091
	50	921.3 ± 21.24	129.7 ± 17.78	0.382 ± 0.017
	55	531.2 ± 6.078	91.05 ± 6.302	0.383 ± 0.007
Starch Rich	45	1180 ± 28.72	225.7 ± 30.04	0.396 ± 0.023
	50	553.0 ± 7.644	40.50 ± 3.301	0.393 ± 0.007
	55	347.3 ± 6.043	24.58 ± 2.485	0.389 ± 0.007

Furthermore, we investigated the dependence of the yield stress and the critical shear rate on the formulation. As shown in Fig. 5(C) and (D) a systematic decay in the *τ*_0_ and the ${\dot{\gamma }}_{\text{cr}}$ is obtained with increasing water content. However, as seen in Fig. 5(C) the yield stress remains in a similar order of magnitudes. Yield stress is critical in printing since it determines the extrudability within the machine limits and the shape fidelity after printing (Lille et al., 2018). With the water content, it is possible to adjust the yield stress and the critical shear rate within the printability limits, without changing the shear thinning response. The following observations are similar to the strain sweep findings where the water content can adjust the *G*_0_ without changing the strain softening response.

Next, we would like to highlight the macronutrient content effects on *τ*_0_ and ${\dot{\gamma }}_{\text{cr}}$. We observe that the *τ*_0_ of the starch-rich systems remain the lowest while fiber and protein-rich samples compete for the highest. Similarly, Fig. 5(C) demonstrates that the ${\dot{\gamma }}_{\text{cr}}$ again appears as the lowest for the starch-rich, and the fiber-rich inks dominate except for the hydration level of 45% WHC. This addresses how macronutrient microstructural network properties cause a slight change in the deformation response.

### 3.3 Comparing the oscillatory and steady rheology indicators

**Fig. 6:**
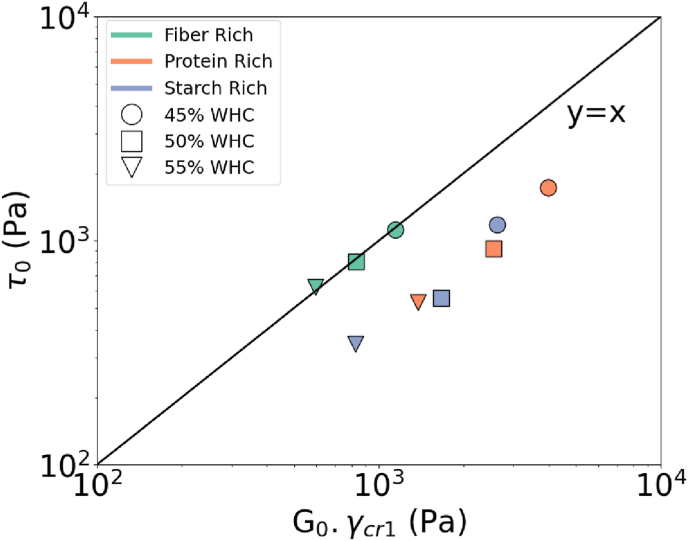
Comparing the *τ*_0_ from steady rheometry with the critical elastic properties from oscillatory rheometry (*G*_0_.*γ*_cr1_).

In this part, the oscillatory and steady methods were compared. First, we focused on the solid-to-fluid transition (SLT) or yielding taking place during the initiation stage of the flow. The concept of yielding simply involves the stress associated with creating an inelastic critical deformation in the material (Kamani et al., 2021). We aimed to capture a similar measure of the yield stress by comparing the indicators from steady and oscillatory rheology. From the shear rate sweeps, we considered the Herschel-Bulkley model parameter *τ*_0_ as one interpretation of the yield stress (Equation (4)). From the strain sweeps, the critical strain was considered as the yielding indicator. We compared the two quantities according to the description in Section 2.3. As observed in Fig. 6 the Herschel-Bulkley model fitted yield stress and the *G*_0_.*γ*_cr1_ are scattered around the identity line.

Considering that the exact value of the yield stress is hard to determine and the recoverability of the deformation at different strain levels is ongoing research for various materials, we think that the correlation between the yield stress of various inks is a promising result for obtaining a characteristic scale for printing applications (Kamani et al., 2021; Dinkgreve et al., 2016). It can be concluded that approximately a factor 1 linear scaling holds for the yield stress from two distinct methods.

**Fig. 7:**
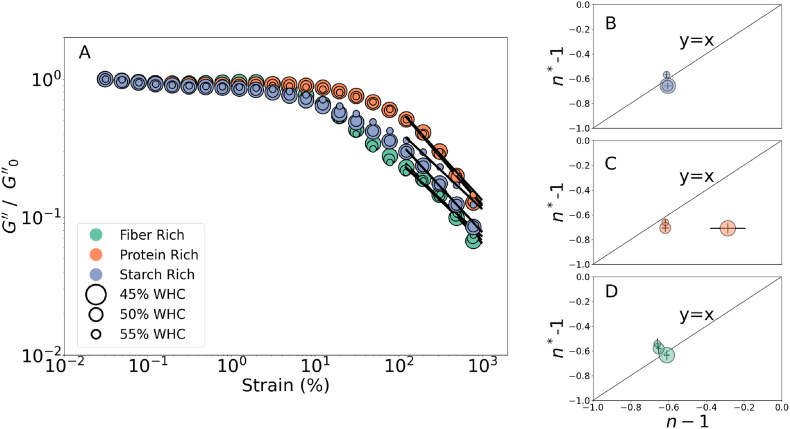
(A) Change of the loss modulus with strain. Black lines indicate the power law model fit. (B-C-D) Comparison of the loss modulus power law exponent (*n*∗ − 1) with the shear thinning exponent (*n* − 1). *G*″/*ω* is normalized by *G*_0_″/*ω* for a clean visual.

Furthermore, shear thinning indices were compared according to the description in Section 2.3, equations (7), (8), (9). Results are shown in Fig. 7. Fig. 7(A) shows the unitless loss modulus with respect to strain amplitude, whereas the black lines indicate the power law description of the last 10 data points. We highlight that one in every 2 data points is plotted in Fig. 7(A) for clarity. Fig. 7(B-C-D) shows the compared shear thinning exponents from the strain sweep and the shear rate sweeps, the unity line is placed to ease the visualization of the values. From the comparison, it can be inferred that values from both techniques ranged between [−0.7,-0.5] except for one outlier being protein at the lowest moisture content. Hence both indicators provide a comparable value, appearing to be independent of the water content and the nutrient composition.

Connections between the steady and oscillatory rheology has been interesting for rheologists. In earlier times, the Cox-Merz rule is utilized by many researchers to extract the shear thinning response from the small amplitude oscillatory testing (Ketz and Graessley, 1988) Furthermore, researchers extended their interest to interpreting the large amplitude oscillatory strain waves to characterize the complex materials and their connection with storage and dissipation-dominated flow properties at pre and post-yielding conditions (Hyun et al., 2002). For instance, the sequence of physical processes approach by Simon Rogers was a big step in the field, connecting the large amplitude oscillatory strain response to the physical processes, giving a better understanding of the elastic, plastic, and viscous effects and their recoverability at certain deformations (Rogers et al., 2011; Rogers and Lettinga, 2012). The approach connecting the oscillatory shearing, steady shearing, and processing flow conditions formed a basis for researchers for a generalized constitutive model formulation for complex materials at high deformations (Ng et al., 2011; Donley et al., 2019; Kamani et al., 2021; van der Sman et al., 2024). Our results show that the steady and oscillatory rheology provides similarity for filled granular pea-pastes; we consider the findings promising for accurate constitutive description in the future.

### 3.4 General discussion: composition and rheology

Table 5 summarizes the findings related to formulation and rheological properties. From a formulation standpoint, the mechanics of microstructural networks play an important role in the rheological properties. To highlight, from the strain sweep analysis, we observed that the LVE region is longer in protein-rich inks further followed by the starch and fiber-rich inks (refer to Fig. 1). An extended LVE region suggests that the structural network formed by the solid contribution remains elastic until a higher strain without any structural alteration (Hyun et al., 2002). Another point of view on this observation from Corker et al. (2019). They claim that the shorter the distance between the deviation and the crossover strain, the more brittle the deformation gets since the change from initiation of the yielding to a complete dissipation state is not gradual but sudden.

**Table 5:** Overview of the rheological parameters with changing composition.

Property	Observation
Strain Sweep	
*G*_0_	Controlled by the water content and the macronutrient ratio
*γ*_cr1_	Controlled by the macronutrient ratio
*γ*_cr2_	Controlled by the macronutrient ratio
*γ*_x_	Independent of the water content and macronutrient ratio
*n*_1_	Independent of the water content and macronutrient ratio
*n*_2_	Independent of the water content and macronutrient ratio
*n*∗	Independent of the water content and macronutrient ratio
Shear Rate Sweep	
*τ*_0_	Controlled by the water content
*n*	Independent of the water content and macronutrient ratio
${\dot{\gamma }}_{\text{cr}}$	Controlled by the water content and the macronutrient ratio
In comparison	
*τ*_0_ and *G*_0_.*γ*_cr1_	Scaled by a factor of 1
*n*_1_ and *n*_2_	Scaled by a factor of 2
*n*∗ and *n*	Scaled by a factor of 1

Another important observation was the order of *G*_0_ following Fiber > Starch > Protein (see Fig. 4). Remarking the tan(*δ*) is scattered around similar values for all the inks so that the inks expected to have a similar initial elastic to viscous ratio under no deformation, this outcome suggests that the fiber-rich inks have a tougher microstructural network which requires more energy input to demolish when compared to the other two.

Also considering that the crossover point is around the same value for all the inks, fiber-rich inks start deviating from linearity at an earlier strain than the protein and starch-rich inks do. This suggests that the strength of the internal network is less strong in the case of fiber-rich inks in comparison with the protein and starch-rich ones. The elasticity of the system starts to demolish earlier strains in the case of fiber-rich inks while it is preserved longer for protein-rich inks, resulting in a more gradual deformation in the case of fiber and a more sudden or brittle deformation in the protein-rich inks.

Starch consists of intact spheres with limited water interactions (according to the low WHC), accordingly, it cannot create a network, but it acts as an inert filler to the molecular matrix created by fiber and the proteins (Venkatachalam et al., 2023). We can further observe the filler effect in the shear rate controlled experiments, where the yield stress and the critical shear rate are below the protein and fiber-rich inks for starch-rich ones (Fig. 1). The increased molecular surface area by the starch spheroids is likely to increase the drag force under a continuous shear rate and allow the system to be sheared easily, as indicated by a lower ${\dot{\gamma }}_{\text{cr}}$.

The literature on the microstructural characteristics supports our rheological observations; Venkatachalam et al. (2023) presented confocal laser scanning microscopy (CLSM) images of pea-based inks rich in fiber and protein. From their pictures, it can be observed that while protein contributed to the network formation by partial dissolution in water, the fibrils of pea fiber do not contribute when used in high concentrations (Venkatachalam et al., 2023). This explains the longer-lasting elastic properties in the case of protein-rich inks in comparison with fiber-rich inks. Similar to their formulations, our inks are essentially highly concentrated suspensions consisting of randomly sized particles where the water-holding properties and the inter-particle interactions of each macronutrient appear to be different. There is a transient network formed by weak interactions such as hydrogen bonding. On top of that in our system, pre-gelled starch acts as the main water absorbent, limits the movement of the free water within the matrix, and contributes to the transient network. Different macronutrients give each formulation a different micro-structural network with a unique deformation response, together with the transient network system formed by the pre-gelled starch. Considering that pea fibrils have a high WHC (refer to Table 1), it can be drawn that they act as absorbent patches with a low resistance to release water. On the contrary, pea protein partially dissolves in the media and contributes to the transient network formation in the continuous phase (Wang et al., 2023).

Upon evaluating the hydration methodology and the WHC hypothesis, we observed that adjusting the fraction of the WHC provided a similar order of magnitude in certain rheological properties. For instance, the order of *G*_0_ and *τ*_0_ remained unchanged across varying water content (Fig. 4, Fig. 5). On top of that the shear thinning (*n* and strain softening indices *n*_1_, *n*_2_) and the crossover strain (*γ*_x_) were independent of the water content within the studied range of percentages of WHC. Highlighting that *G*_0_, critical point, and *τ*_0_ are good indicators of the layer-by-layer building and the *τ*_0_, thinning/softening indices were extrudability markers under ideal flow conditions we can suggest that presumably similar printability and buildability can be obtained via the WHC-percentage-based hydration, according to the similar rheology obtained for the inks.

For the strain sweeps, the same macro nutrient-dominated samples formed a master curve scaled by *G*_0_. Similarly, all the shear rate sweep curves were collapsed on a master curve when normalized by the *τ*_0_. The *G*_0_ and *τ*_0_ are water content dependent within the boundaries of a limited interval, this suggests that the rheology can be scaled with the water content.

By measuring the rheology we have quantified and highlighted the trends in the deformation response of pea-based multicomponent paste-like inks. From a rheological experimentation point of view, inks were quite challenging to measure. The measurements must be completed within a few hours as the sample dries or loses water. During testing, we observed that the edge of the sample, where the sample is interfaced with air, tends to dry the most. Moreover, wall slip and shear banding should be cautiously handled to avoid experimental errors, since it is always been stated as a challenge for highly concentrated suspensions (Fall et al., 2009). Finally, another concept that is important while evaluating the granular material flow is the time-dependent adaptation of the structure to a given shear rate, as known as thixotropy (Coussot, 2020). For us, the time-dependent evolution of the microstructure is challenging to analyze since it was nearly impossible to distinguish the energy lost to the edge fracture and the time effects in larger deformation testing. On one hand, our approach during steady shear rate testing minimized the edge fracture and structural loss to achieve accurate measurement and limit the time spent at the larger shear rates. In that way, we were able to obtain reproducible responses and indicators. But on the other hand, we still find thixotropy as an important concept to investigate in the future, especially for DIW purposes, which involves start-stop cycles during extrusion and post-printing structural recovery. Steady and oscillatory shearing in comparison, opposing the uncontrolled strains in steady deformation rate testing, strain-controlled testing appears to be more practical and reliable. Once it has been translated into the flow indicators, it becomes predictive and can be utilized in ink quality assessment before printing.

It is worth noting that the yield stress and the solid-like structure have to be rebuilt after the extrusion to allow the bottom layers to be suitable for building. This makes recovery of the structure critically important and it needs to be well understood for predictive analysis and accurate constitutive modeling. We hope that the current work will inspire future studies in that direction.

Finally, from a nutritional point of view, results showed that even though macronutrient formulation creates a small difference in the microstructure that further causes variation in deformation response, the rheological characteristics are largely independent of the macronutrient ratio and more dominantly scaled by the water content. To inspire future studies focusing on personalized nutrition, we highlight that this property would provide a guide to obtaining similar textures with different macronutrients. The role of water in the formulation appears to be critical; the rheology and texture can be controlled by the water content for formulations hydrated at around 50% of their water-holding capacity.

## 4 Conclusions

We showed that rheological characteristics such as yield stress and elastic modulus (*τ*_0_ and *G*_0_) of the inks with different functional ingredients shared similar order of magnitudes. Furthermore, we observed *τ*_0_ and *G*_0_ were dependent on the water content. Another observation was that the macronutrients and the water content do not have any effect on the strain softening or the shear thinning properties in the studied range of formulations. In that sense, WHC-adjusted hydration enables manipulation of *τ*_0_ and *G*_0_ without expecting a variation in the strain softening and the shear thinning properties, this confirms that the WHC-based-hydration approach provides similar rheology and plays an important role on the extrudability. Henceforth, water is the major factor in tuning the texture, rheology, and printability of our inks. Macronutrient content created diverse microstructures with a slight variation in breakdown response. This is indicated by the critical strains and shear rates (*γ*_cr1_, *γ*_cr2_, and ${\dot{\gamma }}_{\text{cr}}$) being affected by the changing macronutrient content. Consequently, we anticipate that the macronutrients will also influence the thixotropic behavior of our inks. We note that our preliminary investigations of strain sweeps (low to high, high to low) did indicate thixotropic behavior, with dynamic yield stresses differing from the static yield stresses. It has been discovered that the deformation response of the microstructural network plays an important role in rheological behavior. We compared oscillatory and steady rheology and showed that there is not only a relationship between the yield stress and the end of LVE region (*τ*_0_ - *G*_0_.*γ*_cr1_), but also shear-thinning and strain-thinning indices. This makes it possible to extract the *τ*_0_ without performing steady shear testing for our inks. The relationship between the steady and oscillatory testing techniques, suggests that the deformation response can be captured by the energy dissipation-dominated region of both methods. We successfully took one step closer to personalized nutrition by delving into the effects of water content and the macronutrient ratio on the rheology of multi-component 3D printing inks.

## CRediT authorship contribution statement

**Z.Y. Bugday:** Conceptualization, Methodology, Investigation, Formal analysis, Writing – original draft. **A. Venkatachalam:** Conceptualization, Methodology. **P.D. Anderson:** Conceptualization, Supervision, Writing – review & editing, Funding acquisition. **R.G.M. van der Sman:** Conceptualization, Supervision, Writing – review & editing, Funding acquisition.

## Declaration of competing interest

The authors declare that they have no known competing financial interests or personal relationships that could have appeared to influence the work reported in this paper.

## Data Availability

Data will be made available on request.
